# Temporal Patterns of Honeybee Foraging in a Diverse Floral Landscape Revealed Using Pollen DNA Metabarcoding of Honey

**DOI:** 10.1093/icb/icac029

**Published:** 2022-05-10

**Authors:** Laura Jones, Abigail Lowe, Col R Ford, Lynda Christie, Simon Creer, Natasha de Vere

**Affiliations:** National Botanic Garden of Wales, Llanarthne, UK; National Botanic Garden of Wales, Llanarthne, UK; Molecular Ecology and Evolution Group, School of Natural Sciences, Bangor University, Bangor, UK; Spirent Communications, Positioning Technology, Crawley, West Sussex, UK; National Botanic Garden of Wales, Llanarthne, UK; Molecular Ecology and Evolution Group, School of Natural Sciences, Bangor University, Bangor, UK; Natural History Museum of Denmark, University of Copenhagen, Denmark

## Abstract

Understanding the plants pollinators use through the year is vital to support pollinator populations and mitigate for declines in floral resources due to habitat loss. DNA metabarcoding allows the temporal picture of nectar and pollen foraging to be examined in detail. Here, we use DNA metabarcoding to examine the forage use of honeybees (*Apis mellifera* L.) within a florally diverse landscape within the UK, documenting the key forage plants used and seasonal progression over two years. The total number of plant taxa detected in the honey was 120, but only 16 of these were found with a high relative read abundance of DNA, across the main foraging months (April–September). Only a small proportion of the available flowering genera in the landscape were used by the honeybees. The greatest relative read abundance came from native or near-native plants, including *Rubus* spp., *Trifolium repens*, the Maleae tribe including *Crataegus, Malus*, and *Cotoneaster*, and *Hedera helix*. Tree species were important forage in the spring months, followed by increased use of herbs and shrubs later in the foraging season. Garden habitat increased the taxon richness of native, near-native and horticultural plants found in the honey. Although horticultural plants were rarely found abundantly within the honey samples, they may be important for increasing nutritional diversity of the pollen forage.

## Introduction

Pollination is a key ecosystem service and required for a diverse food supply, with 75% of globally important food crops pollinated by insects (Klein et al. 2007). Due to their ease of management, honeybees are important pollinators in addition to providing humans with honey and wax products (Potts et al. 2016). There is concern, therefore, over honeybee colony loss and ill-health due to increasing pressure from the reduction in the quantity and diversity of suitable foraging habitat coupled with exposure to agrochemicals, apicultural mismanagement, and pest and diseases (Naug 2009; Potts et al. 2010; Goulson et al. 2015). Nutritional stress from the loss and fragmentation of suitable foraging habitat has been suggested as one of the major drivers of colony decline and ill-health (Potts et al. 2010; Wright et al. 2018), meaning an understanding of floral resource use is essential to mitigate habitat declines and maintain healthy honeybee colonies.

Honeybees have been described as super-generalists, however, they have still been shown to be selective in the pollen and nectar they use, even within a diverse floral landscape (de Vere et al. 2017). Despite honeybees being well-studied, there are few studies which examine the plants used by honeybees for foraging, through the active foraging season, which is vital in being able to support colonies throughout their lifecycle. While nectar provides the main energy source, processed into honey for long-term storage, pollen supplies protein, lipids, and micronutrients: crucial for the healthy growth and development of the larvae (Pernal and Currie 2001; Brodschneider and Crailsheim 2010). Different methods have been used to assess honeybee foraging, including direct observation of plants, analysis of waggle dances in the hive (Garbuzov et al. 2015), and analysis of pollen loads and honey to determine botanical composition (Deans 1957; Coffey and Breen 1997).

Traditionally, honey has been characterized using melissopalynology, where pollen is identified morphologically (Coffey and Breen 1997). This requires a high-level of expertize and the level of identification achievable can be limited by a lack of morphological differences in some plant groups (Galimberti et al. 2014). DNA metabarcoding allows for the identification of multispecies samples using high throughput sequencing and has been used to characterize pollen biodiversity for ecological applications (Deiner et al. 2017; Lucas et al. 2018; Brennan et al. 2019; Potter et al. 2019; Lowe et al. 2022a, 2022b). It can be used to efficiently identify the plant composition from pollen in honey (Valentini et al. 2010; Hawkins et al. 2015; de Vere et al. 2017; Smart et al. 2017; Lucek et al. 2019; Jones et al. 2021a; Milla et al. 2022) or from pollen loads (Galimberti et al. 2014; Richardson et al. 2015a, 2021; Danner et al. 2017), reducing the need for time-consuming specialist identification. As with melissopalynology, the capability of DNA metabarcoding in identifying species is only possible with a reference database of the potential species available in the study system, with comprehensive coverage of taxa in the reference database being crucial to the quality of the results (Jones et al. 2021b).

Here, we use pollen DNA metabarcoding of honey to investigate foraging throughout the honeybees’ most active flight period (April—September), over 2 years, using hives set within a complex floral landscape, giving foraging access to both a Botanic Garden and a National Nature Reserve managed as organic farmland. Specifically, we address the following questions.

- Which plants do honeybees use through the year?
- How do the foraged plants compare with the available floral genera?
- What type of plants and habitats are important for honeybee foraging?

## Methods

The National Botanic Garden of Wales study site (62 ha; 51°50“33.4“N; 4°08”49.2”W) was divided into 279 survey zones, circling two apiaries, one contained within the Botanic Garden and one within the National Nature Reserve, sited 1 km apart (Supplementary Figure S1). Each zone was classified into four main habitat types: broadleaved woodland, grassland, hedgerow, and linear features, and garden. For the garden habitat, the survey zones tended to represent distinct flowerbeds, while for the non-planted habitats the zones were split into the main habitat type.

Hives were sampled for honey on a monthly basis from a total of six *Apis mellifera* hives, three at each apiary, from April to September 2016 and 2017. No strongly atypical weather patterns were noted for either year.

To characterize the available plant species for forage, floral surveys were carried out monthly over the same sampling period. Surveys took place over 7–14 days and a list of the plant species in flower (defined as flowers present with available nectar or pollen) was recorded for each zone within the survey area. Plant identification followed Stace (2010) and Rose and O'Reilly (2006). The number of unique genera in flower each month was then calculated for the total survey area.

## DNA extraction

Approximately, 30 ml of honey was collected from each hive using a sterile centrifuge tube, which was crushed against the comb to release the honey. The most recently capped honey was targeted. Any wax was removed using sterile forceps and 10 g of honey was weighed out for DNA extraction using a modified version of the DNeasy Plant Mini extraction kit (Qiagen, Manchester UK; de Vere et al. 2012; Hawkins et al. 2015). Firstly, the 10 g of honey was made up to 30 ml with molecular biology grade water and incubated in a water bath at 65°C for 30 min. Samples were then centrifuged (Sorvall RC-5B) for 30 min at 15,000 rpm, the supernatant was discarded, and the pellet was resuspended in 400 μl of buffer made from a mix of 400 μl AP1 from the DNeasy Plant Mini Kit (Qiagen), 80 μl proteinase K (1 mg/ml) (Qiagen), and 1 μl RNase A (Qiagen). This was incubated again for 60 min at 65°C in a water bath and then disrupted using a TissueLyser II (Qiagen) for 4 min at 30 Hz with 3 mm tungsten carbide beads. The remaining steps were carried out according to the manufacturer's protocol, excluding the use of the QIAshredder and the second wash stage. The OneStep PCR Inhibitor Removal Kit (Zymo Research, Irvine CA) was used to purify the DNA extract. A 1 in 10 dilution of the purified DNA extract was used for amplification.

## PCR and library preparation

A *rbcL* Illumina MiSeq paired-end indexed amplicon library for a 2 × 300 bp kit was created using a two-step PCR protocol, as in de Vere et al. (2017). The samples were first amplified using the template specific primers *rbcL*af and *rbcL*r506 with 5′ overhangs complementary to Nextera XT index primers (Supplementary Table S1). The first PCR had a total volume of 20 μl: 2 μl template DNA, 10 μl of 2x Phusion Hot Start II High-Fidelity Mastermix (New England Biolabs, Ipswich MA), 0.4 μl (0.25 μM) forward and reverse primers, and 7.2 μl of PCR grade water. Samples from this first PCR were assessed by gel electrophoresis on 1% agarose. The first PCR was completed three times and pooled before entering a bead clean up. Thermal cycling conditions were: 98°C for 3 min and 95°C for 2 min; 95°C for 30 s, 50°C for 30 s, and 72°C for 40 s (40 cycles); and 72°C for 5 min and 30°C for 10 s.

The pooled products from the first PCR were then purified following Ilumina's 16S Metagenomic Sequencing Library Preparation protocol using Agencourt AMPure XP beads (Beckman Coulter) with a 1:0.6 ratio of product to beads.

The purified PCR product from round one was followed by a second round of amplification to anneal sample specific Illumina Nextera indices. This index PCR stage used a total volume of 25 μl (12.5 μl of 2x Phusion Hot Start II High-Fidelity Mastermix, 2.5 μl of Nextera XT i7 Index Primer, 2.5 μl of Nextera XT i5 Index Primer, 5 μl of PCR grade water, and 2.5 μl of purified first-round PCR product).

Thermal cycling conditions for the index PCR were: 98°C for 3 min; 95°C for 30 s, 55°C for 30 s, and 72°C for 30 s (eight cycles); and 72°C for 5 min and 4°C for 10 min. The index PCR product was then purified following the PCR clean-up 2 section of the Illumina protocol, using a 1:0.8 ratio of product to AMPure XP beads.

The purified products of the index PCR were quantified using a Qubit 3.0 fluorometer (Thermo Fisher Scientific, Loughborough, UK) and pooled at equal concentrations, producing the final library. Negative controls using PCR grade water were amplified and sequenced alongside honey samples. Sequence data is available at the SRA under the BioProject number PRJNA748230.

## Bioinformatic analysis

Sequence data were processed using a modified bioinformatic analysis pipeline first developed in de Vere et al. (2017;https://github.com/colford/nbgw-plant-illumina-pipeline). Raw reads were trimmed to remove low quality regions (Trimmomatic v. 0.33), paired, and then merged (FLASH v. 1.2.11), with merged reads shorter than 450 bp discarded. Identical reads were dereplicated within samples and then clustered simultaneously at 100% identity across all samples (vsearch v. 2.3.2), with singletons (sequence reads that occurred only once across all the samples) then discarded.

A custom reference database was created for sequence identification. A species list containing 5586 species was generated using the list of native species of the UK (Stace 2019), naturalized and alien species (Preston et al., 2002), planting records from the IRIS BG horticultural database at the National Botanic Garden of Wales, and survey data records from the 2016 and 2017 floral surveys. All available *rbcL* plant records were downloaded from NCBI GenBank, including the Barcode UK reference library (Jones et al. 2021b). The total species list was used to extract relevant records using the script *creatingselectedfastadatabase.py* (available at https://github.com/colford/nbgw-plant-illumina-pipeline). For plants on the species list not represented at species level within GenBank, a second extraction was completed for records at the genus level. In the reference database created, species level coverage for *rbcL* was 57%, and coverage at genus level was 96%.

The sequence data from the honey samples were compared against the reference database using blastn, using the script vsearch-pipe.py. The top 20 BLAST hits were then summarized. If the top bitscore of a sequence matched to a single species, then the sequence was identified to that species. If the top bitscore matched to different species within the same genus, then the result was attributed to the genus level. If the top bitscore belonged to multiple genera within the same tribe or family, then a tribe or family level designation was made. Sequences that returned families from different clades were considered to be chimeric and excluded. These computed identifications were then checked manually for botanical veracity, in terms of the phenology of the plants and their presence within the study site.

## Assigning traits to plant taxa

The native status, form, and habitat were assigned for all plant taxa identified at genus and species level with the DNA metabarcoding. Taxa identified at family level were not categorized. The native status of the identified taxa was assigned to one of three categories: native or near-native, naturalized, and horticultural. The native or near-native category includes native plants and archaeophytes to the UK as defined by Stace (2019), in addition to near-native genera that can represent both native species and horticultural varieties in the study site, which are ecologically similar, i.e., non-native species that had a native representative in the genus. Naturalized taxa represented plant species which are widespread and self-perpetuating in the wild. All remaining taxa were categorized as horticultural.

The growth form of the plant taxa was assigned to either tree (woody species > 5 m), shrub (woody species < 5 m), or herb (non-woody species). The plant taxa identified in the honey were matched to the categories of habitat recorded in the survey site. The habitat type categories were (a) broadleaved woodland, (b) grassland, (c) hedgerow and linear features, and (d) garden. Garden habitat represented areas of the Botanic Garden which are planted, including native and non-native plant species, alongside horticultural taxa. Grassland habitats included both semi-improved grassland and species-rich meadows, either managed by grazing or cutting. Hedgerow and linear features included hedgerow habitats and scrub field margins.

The plant taxa found in the honey were categorized into four measures of abundance according to the relative read abundance of DNA sequences found in each month: those representing over 10% of sequences were designated as major plant taxa, between 1 and 10% secondary taxa, between 0.01 and 1% minor taxa, and below 0.01% were occasional.

### Statistical analysis

The change in honey plant composition over the 2 years was examined using a generalized linear model, using the “manyglm” function in the R package *mvabund* (Wang et al. 2012). The data best fit a negative binomial distribution, with the large number of zero values for taxa across the samples resulting in a strong mean–variance relationship. The multivariate response variable was the abundance table of plant taxa (number of reads), with the variable sequencing depth between samples controlled for by including the total number of reads per sample as an offset in the model. Model assessment was based on the Akaike information criterion score and inspection of the residuals (Supplementary Table S2 and Supplementary Figure S2). Month and year were included as predictor variables to examine the effect of time. Samples collected in April 2017 were excluded from the model analysis to allow comparison between the sampling years.

To examine the change over time in proportion of reads attributed to plant status, form, and habitat, three generalized linear models were run using the “manyglm” function. The multivariate response variable was the abundance of reads assigned to each plant trait category for each sample, with variable sequencing depth controlled for with the total number of reads per sample included as an offset in the model. Model assessment was based on the Akaike information criterion score and inspection of the residuals (Supplementary Table S2). The best model for plant status and form included month as a predictor variable, while the model for habitat used both month and year.

Non-metric multidimensional scaling (NMDS) ordination was used to visualize monthly changes in the composition of the honey, based on the proportion of reads returned for each plant taxa. Ordinations were carried out using the metaMDS function in the R package *vegan* (Dixon 2003) using Bray–Curtis dissimilarity indices. All statistical analyses were performed using R 4.0.3 (R Development Core Team 2011).

## Results

Across the survey period, a total of 66 honey samples were collected. Honey could not be collected in April 2016 due to a lack of available stored honey. The *rbcL* sequencing run yielded a total of 11,916,038 returned read pairs. After all quality control steps 6,688,579 sequences were taken forward for analysis. Of the 66 honey samples, three returned less than 100 sequences and were excluded from further analysis. The mean sequence number returned for each sample was 106,525 (SD = 42,025) and ranged from 33,971 to 217,408.

In total, 120 plant taxa were identified across all 63 samples. Of the sequencing reads returned, 26% were assigned to species, 60% to genus, 8% were matched to a tribe, and 6% to family. When examining the overall abundance of taxa identified, only 17 plants were returned at over 1% of all sequences.

### Which plants do honeybees use through the year?

**Fig. 1:**
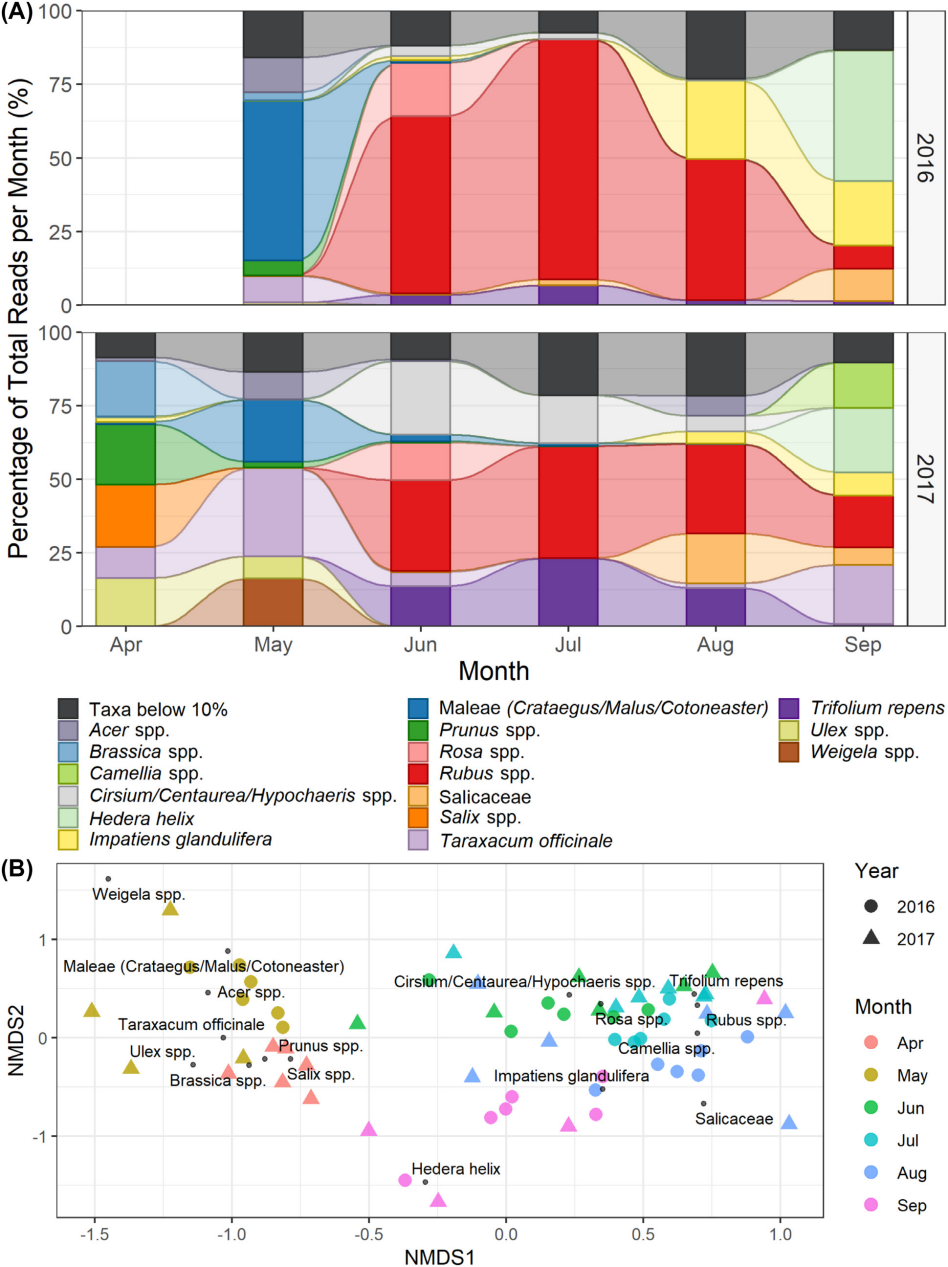
(**A**) Plant taxa identified from 63 honey samples. Taxa labeled represent over 10% of sequencing reads returned for at least 1 month, described as major floral resources. (**B**) NMDS ordination of the honey samples collected over 2 years. Color indicates the month of collection and shape indicates the year. Plant taxa found in over 10% of the reads for each month are plotted separately indicating the major taxa driving the changes each month on community composition of the honey

When examining on a monthly basis across the 2-year period sampled, only 16 taxa were found at over 10% of the sequences returned in at least 1 month, these were categorized as the “major” floral resources (Fig. 1 and Supplementary data). The plant composition of the honey changed significantly through the foraging season: by the month the honey sample was collected (Fig. 1: LR = 1376, *P* = 0.001), along with the years (2016 and 2017) of sample collection (Fig. 1: LR = 303, *P* = 0.001). NMDS ordination (Fig. 1B) supports that the samples from each month are most similar to each other, with increasing dissimilarity as the season progresses.

In April, *Salix* spp., *Prunus* spp., *Ulex* spp., and *Brassica* spp. were the major plants used by the honeybees. In May, the use of the Maleae tribe representing *Crataegus, Malus*, and *Cotoneaster* spp. became more abundant, along with *Taraxacum officinale, Acer* spp., and *Weigela* spp. (Fig. 1A).

Moving from spring to summer shows a shift in the major plants used by the honeybees. For both years, *Rubus* spp. was the top taxon found in June, July, and August. Additional major forage plants in use by the honeybees through these months are*Trifolium repens, Cirsium/Hypochaeris/Centaurea* spp., *Rosa* spp., and the willow family Salicaceae. *Impatiens glandulifera* was first used as a major forage in August. Finally, in September, the autumn-flowering *Hedera helix* appeared as a major forage in both years, alongside *I. glandulifera* in 2016 and *Camellia* spp. in 2017.

### Annual variation in forage

While the majority of the major taxa were identified in both years, there was variation in their relative abundance when comparing months between years (Fig. 1A). In total, five of the 16 taxa were used consistently as major forage in both years: *Rubus* spp., the *Crataegus, Malus, Cotoneaster* spp. group, *H. helix*, a member of the Salicaceae family, and *Rosa* spp. A total of eight taxa were classed as major in 1 year while appearing as secondary forage (1–10% of sequencing reads returned in a month) in another. For example, *T. repens*, a major taxon in 2017, was secondary forage in 2016 where the months in which it appeared were mostly dominated by *Rubus* spp. The remaining two taxa, *Salix* spp. and *Ulex* spp. were only major forage in April 2017, a month not sampled in 2016.

**Fig. 2:**
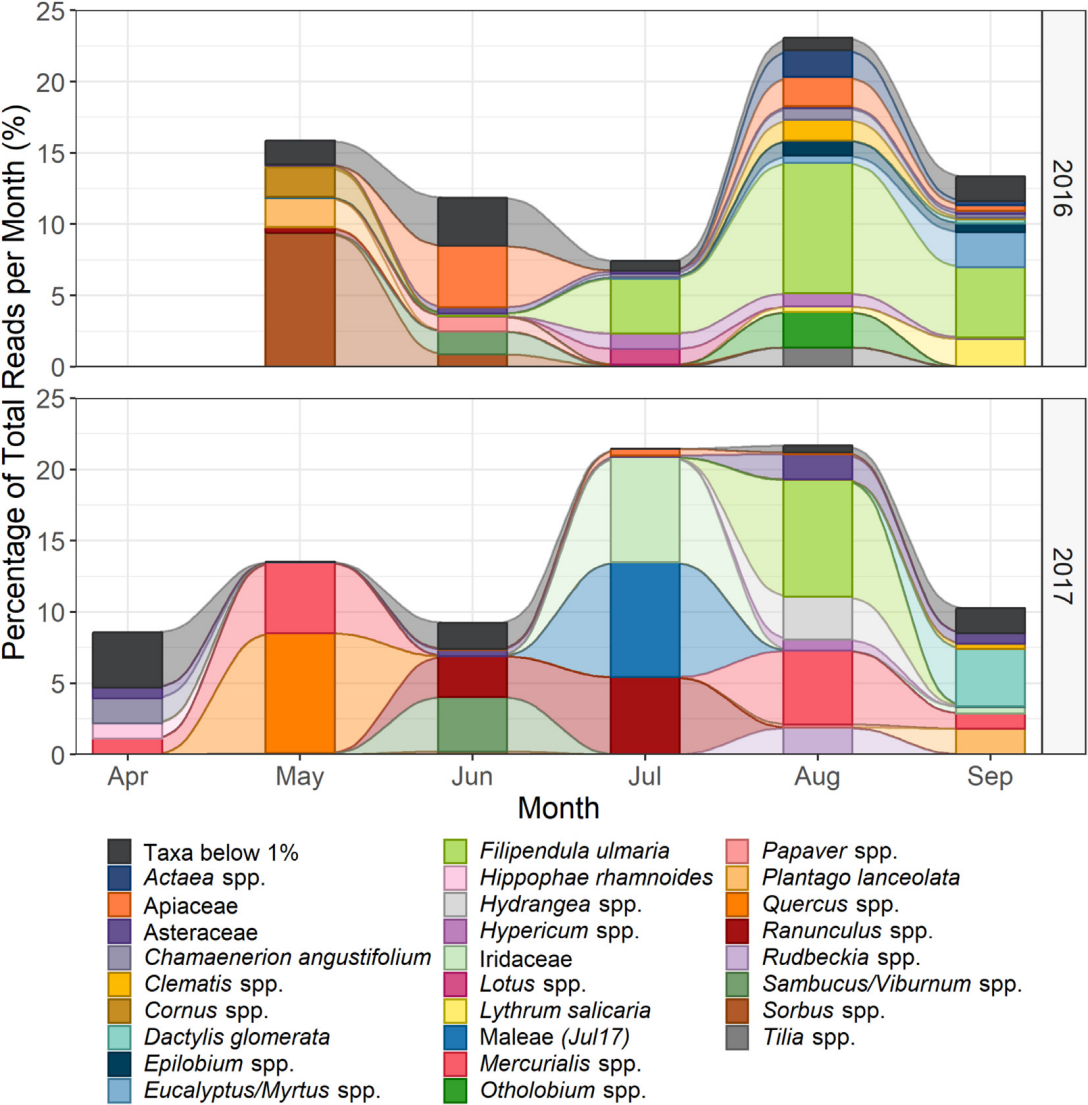
Plant taxa identified from 63 honey samples. Taxa labeled represent between 1 and 10% of sequencing reads returned for at least 1 month, described as secondary forage sources

There was a greater difference between the years for the 27 plant taxa designated as secondary forage (Fig. 2), with only three of the 27 plants considered secondary forage for both years: *Filipendula ulmaria*, the *Sambucus*, and *Viburnum* spp. grouping, and *Plantago lanceolata*. The *Sambucus* and *Viburnum* spp. grouping was found consistently in June, while *F. ulmaria* was found in July, August, and September for 2016 and mostly found in August for 2017. *Plantago lanceolata*, which flowers throughout the season, was particularly distinct between the 2 years being found in May 2016 and September 2017.

### Comparison with floral resources available in the landscape

Across the two foraging years, 561 unique genera were recorded in flower within the study site. A total of 90 genera were found in the honey with 89 of these recorded within the survey area, with the sole exception of *I. glandulifera*. When comparing on a monthly basis the plant genera used with the available flowering genera, it shows that the honeybees were only using a small proportion of what was available through the season (Table 1). April 2017 had the highest proportion of plant genera available that were also found in the honey with 22%, whilst the lowest was July 2017 with 6% of genera available detected as in use by the honeybees.

**Table 1:** Plant genera detected in the honey compared to availability within the study site. For each month, generic richness is noted and compared with the flowering availability (presence/absence). Only a small proportion of the plant genera available were ever detected in the honey

	2016					2017					
	May	Jun	Jul	Aug	Sep	Apr	May	Jun	Jul	Aug	Sep
Number of genera in flower in the study site	237	310	278	261	224	148	215	275	252	247	238
Number of genera found in honey	41	40	30	37	40	50	18	37	23	35	26
Genera found in honey and flowering within the study site	37 (90%)	33 (83%)	24 (80%)	25 (68%)	28 (70%)	33 (66%)	16 (89%)	28 (76%)	16 (70%)	26 (74%)	21 (81%)
Proportion of genera in flower used by honeybees	16%	11%	9%	10%	13%	22%	7%	10%	6%	11%	9%

At low levels, honey samples contained taxa which were known to be flowering in previous months. For example, in the April honey samples 4% of reads returned could be attributed to taxa flowering in previous months, including 10 late-flowering plants which were also detected in September 2016: *H. helix, I. glandulifera, Rubus* spp., *F. ulmaria, Lythrum salicaria, Campsis* spp., *Actaea* spp., *Dactylis glomerata, Chamaenerion angustifolium*, and *Oenothera* spp.

### Relationship to the native status, form, and habitat of plant taxa

**Fig. 3:**
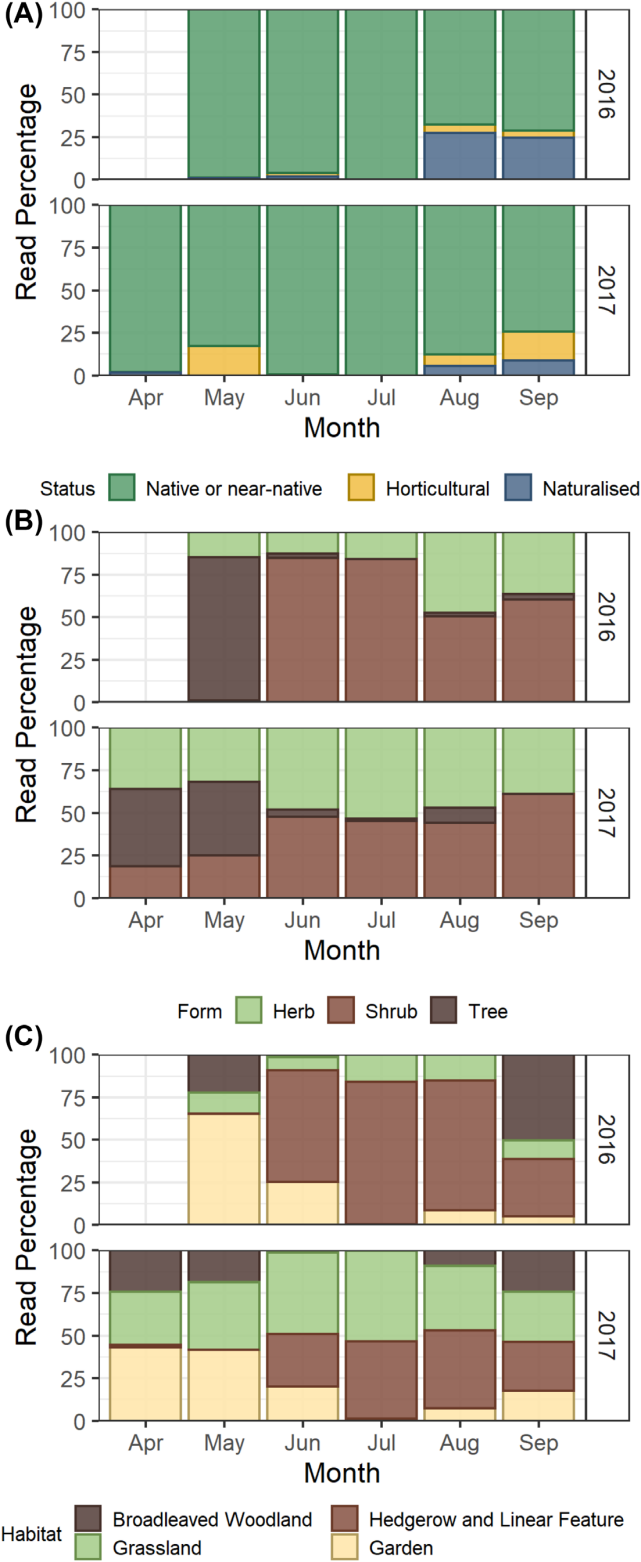
Proportion of sequences returned, characterized by plant traits, (**A**) native status, (**B**) growth form, and (**C**) main habitat. Taxa returned at family level were excluded

Taxa designated at species, genus, and tribe level from the DNA results (101 out of the 120 total taxa recorded) were classified according to the status, form, and habitat of the plant (Supplementary Data). Native or near-native plants represented 68 of the taxa and 83% of total reads returned, 30 taxa were horticultural plants representing 4% of reads, and there were three naturalized taxa returning 6% of total reads (Fig. 3). Horticultural and naturalized species, including *I. glandulifera, Camellia* spp., and *Eucalyptus/Myrtus* spp., contributed most toward the end of the season in August and September, with the proportion of taxa by status changing significantly over the sampled months (LR = 44.28, *P* = 0.001). *Weigela* spp. represented the most abundantly foraged horticultural genus in May 2017 (Fig. 3A).

For plant form, overall, 44% of all sequences returned were shrubs, representing 27 taxa, 32% were herbs with 55 taxa and 16% were trees covering 19 taxa. Trees were used mostly in the early foraging season of April and May (Fig. 3B), and comparatively much less as the season progressed, where foraging was split between herbs and shrubs. These seasonal progressions significantly differed by month (LR = 38.10, *P* = 0.002). Spring flowering tree genera used by the honeybees included *Salix, Prunus, Acer, Sorbus, Quercus*, and *Cornus*.

When examining the main habitat of the plants recorded in the honey overall, hedgerow and linear features accounted for 36% of the total sequences returned but this represented only four plant taxa, the major taxa *Rubus* spp. and *I. glandulifera*, followed by *Convolvulus/Calystegia* spp. and *Silene* spp. at lower levels. Grassland habitat was associated with 25% of reads and 22 taxa, broadleaved woodland was 12% of reads and 12 taxa, while garden habitat was 19% of reads representing 63 different taxa within the landscape. While horticultural taxa contributed most toward the end of the season (Fig. 3A), garden habitat containing plants such as the native or near-native *Prunus* spp. contributed most in spring (Fig. 3C). The habitat of the plant taxa used changed significantly over time for both month (LR = 120.31, *P* = 0.001) and year (LR = 12.83, *P* = 0.015), reflecting the biggest percentage change, which was in the increased use of plant taxa from grassland habitat between 2016 (6%) and 2017 (20%).

## Discussion

The honeybees within this study had access to a high diversity of plants, both native and horticultural but only used a small proportion of the floral resource available. The seasonal changes in composition of the plant taxa found within the honey significantly varied by month and year. DNA metabarcoding provided a method for detailed phenological assessment of the honey and tracked with the known flowering phenology of the plants. However, how accurately DNA metabarcoding represents the relationship between the abundance within the system and the abundance of sequences returned is debated and has been referred to as semi-quantitative (Keller et al. 2015; Richardson et al. 2015b; Bell et al. 2019; Deagle et al. 2019).

In de Vere et al. (2017), honeybee foraging in April and May 2015 was characterized using DNA metabarcoding within the same study site. The results presented here are consistent with these spring results, continuing the trend that honeybees use a small percentage of the total genera available to them through the season. While the plants used are taxonomically diverse, there are a small number of core species which form the majority of the honeybee diet. Honeybees have been referred to as super-generalists within plant–pollinator networks, however, as evidenced here, they are still using a selection of plants within a system and not using everything available to them. Similarly, in a meta-analysis of plant-pollinator interaction networks, frequent honeybee visitation was found to be restricted to a minority of plant species (Hung et al. 2018). The plant species other generalist and specialist pollinators are selecting within the system is, therefore, a key area for further research, to establish a full understanding of the pollinator–plant assemblies. While it is important to provide honeybees with a diversity of forage to ensure nutritional variety and contingency against environmental variation in nectar and pollen availability, any siting of hives should also consider their access to the highly abundant species which they frequently target.

Month was found to have the biggest effect on the plant taxa composition of honey. Similar results in terms of the abundance of the major plants found within the honey and foraging phenology have been seen in studies in the UK and Ireland using microscopic techniques to identify the pollen in honey and pollen loads (Percival 1947; Deans 1957; Coffey and Breen 1997), as well as when examining honey samples using DNA metabarcoding of markers *rbcL* and ITS2 from across the UK (Jones et al. 2021b). In Coffey and Breen (1997), plants identified from the pollen found within freshly collected nectar from hives in Ireland showed similar spring foraging patterns, with *Salix* spp. *Ulex* type and *Prunus/Pyrus* type identified using pollen morphology in April and May. *Trifolium repens* and *Rubus* spp. were the main providers of nectar and pollen in June and July, supplemented with *F. ulmaria*. Later in the season, Coffey and Breen (1997) also identified *I. glandulifera, Calluna vulgaris*, and *H. helix* as locally abundant sources. *Calluna vulgaris* was not identified in the honey here, despite being present at low quantities within the survey area, but the other plants found are highly consistent with our findings.

The flowering phenology of the plants found in the honey matched that of the survey area well, with spring species found abundantly in the April–May samples (e.g., *Salix* spp., the Maleae tribe, *Crataegus* spp.*, Malus* spp., and *Cotoneaster* spp.), and key late-flowering species abundant in the September samples (e.g., *H. helix*and*I. glandulifera*). However, species associated with different seasons were found at lower levels in different months, most notably in April 2017: where low levels of *H. helix* and *I. glandulifera* can be explained by the carried over presence of honey stores from the previous year. Honey as a record of foraging covers a longer time period than pollen present on individual foragers, with pollen potentially remaining present within the stores over a longer time period than a month. Here, we targeted the most freshly capped honey, however, honeybees may relocate honey stores within the hive (Eyer et al. 2016).

Across all the months, *Rubus* spp. accounted for 30% of the total reads returned, with *T. repens* second at 7%. *Rubus* spp. have a long flowering period, with *Rubus fruticosus* flowering from June until September (Gyan and Woodell 1987) and *T. repens* covering a similar flowering period (Burdon 1983). A similar pattern was seen in a DNA metabarcoding survey of 441 UK honey samples, where *Rubus* spp. was the most frequently identified taxon, present in 73% of samples, followed by *T. repens* which was present in 62% (Jones et al. 2021a). *Trifolium repens* represents one of the largest potential nectar sources within the UK, as a component of agricultural grasslands, although it has declined in abundance due to changes in agricultural practices (Baude et al. 2016; Jones et al. 2021a).

The year of sampling was also found to have a significant effect on the plant composition of the honey, indicating the importance of multiple years of sampling to build a fuller picture of the seasonality of forage. The plants identified as major taxa were more consistently present between the years, with shifts in their relative abundance within the honey. There was more variety in the occurrence of plants used at a lower level. Many environmental factors, such as temperature, humidity, and precipitation will affect the availability and quality of both nectar and pollen within the plant (Corbet et al. 1979; Corbet 2003; Nicolson et al. 2007), which could lead to variation in the reward for a forager between seasons. The weather conditions can also influence the level of honeybee foraging. A period of low temperature and/or high precipitation can prevent the honeybee foragers from leaving the hive and can result in plants with a short flowering period being missed completely. For example, among beekeepers, *Crataegus monogyna* is considered inconsistent in the nectar flow offered for honey production over different years (Howes 1945). It also has a shorter flowering period compared to other woody Rosaceae species, making it more vulnerable to being missed by the honeybees due to inclement weather (Gyan and Woodell 1987). Here, the Maleae tribe was abundant in the landscape during both years in May, but only remained as secondary forage through to June and July in the 2017 season.

While the importance of garden habitat to pollinators has been shown in urban areas (Baldock et al. 2019) and areas which are intensively farmed (Samnegård et al. 2011), we found that in a landscape with horticultural, semi-natural, and native habitats, native and near-native plants made up the majority of taxa used by the honeybees. When comparing between trial plots with native, near-native, and exotic plants, Salisbury et al. (2015) found that a greater floral resource resulted in an increase in pollinator visits, with a greater abundance of pollinators on native and near-native plants compared with exotic plants. Here, we find that horticultural species are used at low levels through the season. Within the wider landscape of primarily native and agricultural habitats, the taxa found in garden habitats may be increasing the diversity of species used by the honeybees, including both native or near-native plants and horticultural plants. Diversity of pollen diet has been linked to increased immunocompetence in honeybees (Alaux et al. 2010; Di Pasquale et al. 2013), and horticultural taxa and garden habitat can increase the available diversity, although not necessarily abundance, of forage on a landscape scale.

The hedgerow and linear features habitat contributed a disproportionate abundance of forage compared to the area covered in the study site, driven by *Rubus* spp. Hedgerows have been named as a potential way to efficiently increase the available nectar in a landscape, due to their high nectar productivity within a small area (Baude et al. 2016).

The honey was found to contain more tree taxa in spring compared with later in the season, consistent with spring foraging patterns found in de Vere et al. (2017). A similar pattern is seen in Balfour et al. (2018) with UK insect-pollinated plant species classified into trees, shrubs, and herbs, with trees peaking in spring, shrubs in early summer, and herbs in July. Beekeepers have noted a “June gap” where the availability of nectar from native floral sources is said to be lacking (Percival 1947; Crane 1976; Coffey and Breen 1997; Timberlake et al. 2019). Balfour et al. (2018) highlighted that this June gap may be occurring in between the flowering peaks of tree and herbaceous plants. The spring tree species found here, such as *Salix* spp., *Acer* spp., *Quercus* spp., and *Cornus* spp. are key to providing pollen, vital to the healthy growth and development of the larvae and the colony early in the season (Brodschneider and Crailsheim 2010).

Here, the foraging of honeybees was examined in a diverse landscape over multiple years, providing a detailed phenological examination of the forage, vital to evidencing the plants most important to supporting hives. Using DNA metabarcoding, we were able to discover the plants that are most important to honeybees through the foraging season. Time, and therefore, floral phenology, significantly affected the composition of plants used by honeybees. The major forage plants found in the honey through the season were characterized by being native and near-native plants, often found in hedgerow and linear features and grassland habitats. Tree genera, found in broadleaved woodland, were an important source of spring forage, followed by herbs and shrubs through to summer and autumn. The plants foraged at a lower level included horticultural plants. There are implications both for the management of habitat in the landscape for honeybees and the siting of hives. While the horticultural plants may be supplying the honeybees with the floral diversity that they require, any high quantity of hives should be placed considering their access to the semi-natural and native habitats which supply the majority of their diet.

## Authorship

The study was conceived by N.d.V. and L.J. The lab work was carried out by L.J., field work was carried out by L.J., A.L., and L.C. The data were compiled by L.J. and A.L. and analyzed by L.J., C.F. with suggestions from N.d.V. and S.C. The manuscript was written by L.J. and N.d.V. with contributions from all of the authors.

## Supplementary Material

icac029_Supplemental_File

## Data Availability

The data underlying this article are available in the article and in its online supplementary material and in the Sequence Read Archive (SRA) under the BioProject number PRJNA748230.

## Notes

From the symposium “DNA metabarcoding across disciplines: sequencing our way to greater understanding across scales of biological organization’’ presented at the annual meeting of the Society for Integrative and Comparative Biology virtual annual meeting, January 3–February 28, 2022.
