# Supplementary material for: Temporal Patterns of Honeybee Foraging in a Diverse Floral Landscape Revealed Using Pollen DNA Metabarcoding of Honey
Source: Integr Comp Biol. 2022 May 10;62(2):199–210. doi: 10.1093/icb/icac029 (PMC9405717; doi:10.1093/icb/icac029)
Supplement: icac029_Supplemental_File [file icac029_supplemental_file.zip › icb-2022-0018-File006.docx]

**Supplementary Table 1.** Metabarcoding primer details, with Illumina overhangs and template specific primers.

| Primer name | Universal Tail | Template specific primer | Reference |
| --- | --- | --- | --- |
| *rbcL*af  (Forward) | TCGTCGGCAGCGTCAGATGTGTATAAGAGACAG | ATGTCACCACAAACAGAGACTAAAGC | (Kress and Erickson, 2007) |
| *rbcL*r506  (Reverse) | GTCTCGTGGGCTCGGAGATGTGTATAAGAGACAG | AGGGGACGACCATACTTGTTCA | (de Vere et al. 2012) |

de Vere, N., Rich, T.C.G., Ford, C.R., Trinder, S.A., Long, C., Moore, C.W., Satterthwaite, D., Davies, H., Allainguillaume, J., Ronca, S., Tatarinova, T., Garbett, H., Walker, K., Wilkinson, M.J., 2012. DNA Barcoding the Native Flowering Plants and Conifers of Wales. PLoS ONE 7, e37945.

Kress, W.J., Erickson, D.L., Jones, F.A., Swenson, N.G., Perez, R., Sanjur, O., Bermingham, E., 2009. Plant DNA barcodes and a community phylogeny of a tropical forest dynamics plot in Panama. Proceedings of the National Academy of Sciences 106, 18621–18626.

**Supplementary Table 2.** AIC scores for all models.

| **Model** | **Model Variables** | **AIC sum (*manyglm*)** | **ΔAIC** |
| --- | --- | --- | --- |
| All taxa | Month + Year | 17492.23 | Min |
|  | Month | 17555.50 | +63.27 |
|  | Year | 17657.39 | +101.89 |
|  | Month + Year + Month:Year | 18258.61 | +601.22 |
| Plant Native Status | Month | 2626.455 | Min |
|  | Month + Year | 2630.609 | +4.154 |
| Plant Form | Month | 3493.839 | Min |
|  | Month + Year | 3495.298 | +1.459 |
| Plant Habitat | Month + Year | 4326.784 | Min |
|  | Month | 4331.612 | +4.828 |


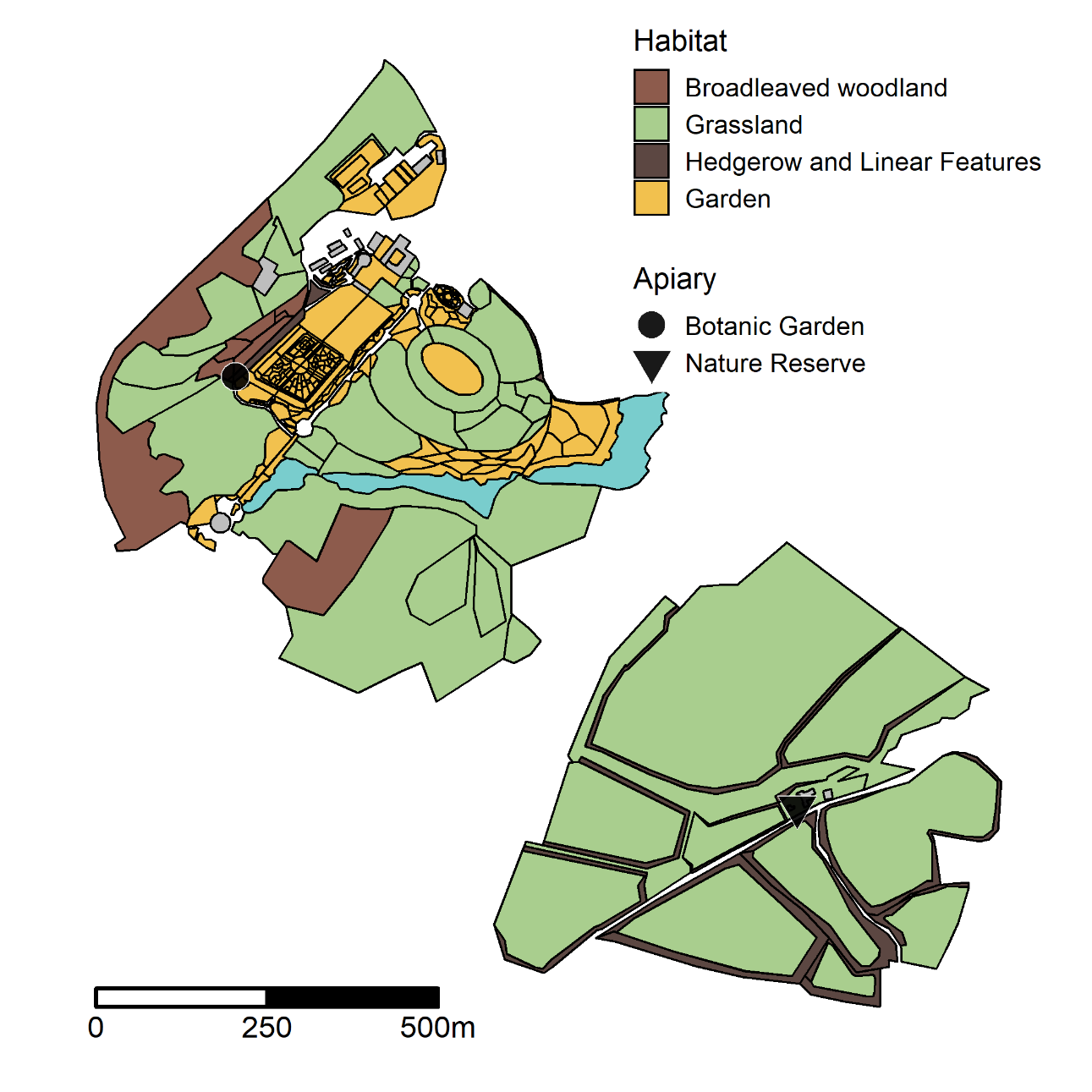


**Supplementary Figure 1:** Illustrating the different close-range habitat types within the survey area and the two apiaries set in the study site. Grassland consisting of predominantly semi-improved natural grassland is the greatest area covered. Maps were created in QGIS v 3.6.1 and R v 4.03 from OS data © Crown Copyright (2018) licensed under the Open Government Licence.


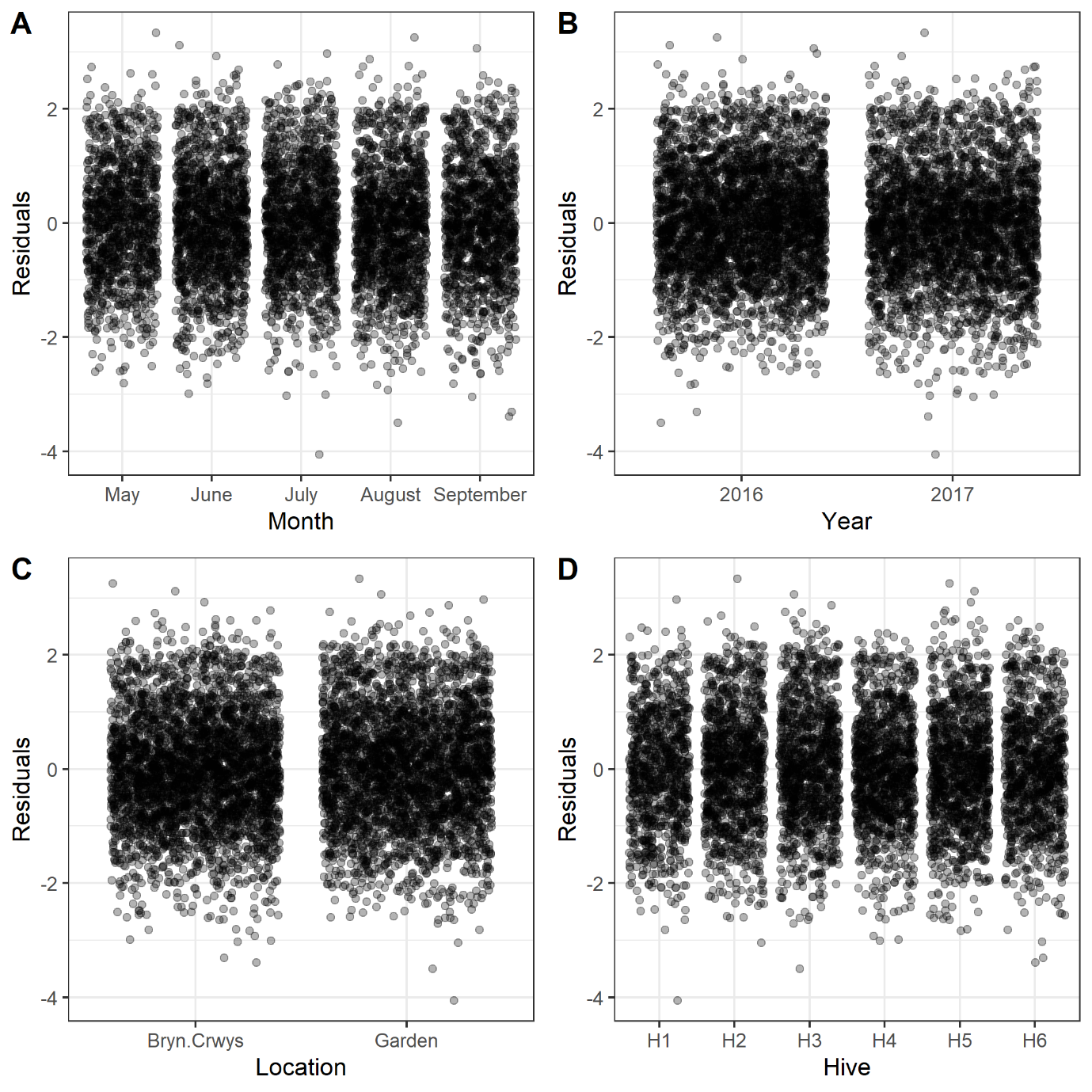


**Supplementary Figure 2.** Scatter plot of the residuals from the model using abundance data of all plant taxa from DNA metabarcoding and the variables a) month of sampling, b) year of sampling, c) location of the apiary d) hive sampled.
